# From the Service of Prof. N. S. Davis, in Medical Wards of Mercy Hospital

**Published:** 1868-06

**Authors:** 


### FROM THE SERVICE OF PROF. N. S. DAVIS,

*In Medical Wards of Mercy Hospital.*

### EPILEPSY.—From Notes by —. Barstow. *April 8, 1868.*

Gentlemen:—The patient before you is a woman aged 45 years; the mother of several children. The countenance has a slight expression of sadness, coupled with sufficient paleness to indicate some deficiency of red-corpuscles in the blood, though no marked emaciation. She represents her appetite as fair; her circulation and respiration are undisturbed; and her secretions and excretions are represented as natural. But she has been subject to paroxyms of epilepsy, or falling sickness as it is sometimes called, during the last three years. With her the paroxysm comes suddenly, without any premonitory symptom or peculiar warning. The head begins to swim with dimness of vision, and in a very few seconds, she falls unconscious, in whatever place she may be standing or sitting at the time. The fall is accompanied by general muscular spasms, contorting the features, suppressing respirations, and stiffening the muscles of the trunk and extremities, until the face becomes very turgid and purple from the unoxygenated blood. In a few moments the spasmodic action ceases, the respirations are resumed, at first very irregularly with the forcible ejection of saliva often mixed with blood from the biting of the tongue or lips, and afterwards more regular and quiet like one in sleep. This period of quiet or apparent sleep continues from fifteen to thirty minutes, when she awakes with an expression of surprise coupled with a feeling of weariness, but is soon able to go about as usual. She usually has one or two paroxyms each day for two days in succession, and then is exempt from their return for 12 or 14 days. The marks of recent injury which you see on her forehead and nose were produced by falling in one of her paroxysms just before her admission into the hospital some four days since, and these extensive cicatrices on her forearms, were occasioned by her falling upon the hot stove in her family several months ago. The frequent repetition of her paroxysms for three years has perceptibly impaired her mental faculties, including the memory. The failure of the last named faculty is the first to be noticed by the friends of the patient.

She says her family often "*scold* her" for forgetting everything. This case will illustrate one of the most common forms of epilepsy originating in adult life.

Here is a case from another ward in the hospital. It is a man near 40 years of age, who has had epileptic paroxysms ever since he was 14 years old. You see in his expressionless face and undeveloped forehead, unmistakable evidences of imbecility and impaired cerebral nutrition. Now, when he should be in the vigor of manhood, he is a mere indolent child, caring for nothing but food and tobacco. The nature or pathology of epilepsy is involved in much obscurity. It occurs in both sexes, and at all periods of life, though it more frequently commences in childhood and youth. Its paroxysms exhibit every degree of severity, from mere momentary giddiness and arrest of mental action to the most violent and general convulsions. They may vary from once in one or two years to five or six times per day. The disease affects persons of widely diverse temperaments or physical conditions, and may be excited by a great variety of causes. The latter, however, may be divided into two classes, *viz.*: those that establish the primary seat of irritation in some part of the periphery or sentient extremities of the nervous system, and these that act more directly on the brain or cerebro-spinal nervous centres. Hence many modern writers have divided all cases of epilepsy into two classes: the one called centrifugal or peripheral, and the other centripetal or concentric. To the first class belong such cases as arise from primary irritation in the alimentary canal; in the uterus; in the sexual organs of both sexes, more especially from masturbation and excessive sexual intercourse; and in the wounded or mechanically injured nerves of the extremities or any part of the body. To the second class belong those cases that arise from causes acting directly on the brain or nervous centres; such as mechanical injuries of the brain, depressing mental emotions and passions, alcoholic and other cerebral exhilarants, etc. None of the causes belonging to either of the classes mentioned, are sufficient to induce epilepsy without the preëxistence of a peculiar morbid excitability, of the cerebro-spinal centres favorable to the development of spasmodic paroxysms. This morbid excitability is indeed the only pathological condition that appears common to all cases of epilepsy.

This, with the special physical condition of the patient, and the nature of the exciting cause, must determine the indications for treatment in each case. To ferret out and remove the exciting cause, more especially in those cases classed as *peripheral*, is a step of primary importance. To expect a cure of the disease by any one of the supposed specific or empirical remedies, while the reflex influence of a dysmenorrhœa, a gastric or intestinal irritation, over-excited genital organs, or an injured nerve, is constantly radiating a morbid impression upon the nervous centres, would be unreasonable. After due attention to all matters of this class, the next step is to regulate the diet, exercise, and mental habits of the patient. In all cases, except such as exhibit evidences of positive impoverishment of the blood and tissues, I think it important to exclude meat altogether as an article of food. Milk, farinaceous articles, tuberous roots, and fruits may be taken freely. In all cases, all kinds of fermented or distilled spirits, together with *strong* tea and coffee should be rigidly avoided. It is more safe to wholly exclude tobacco also. Moderate daily exercise in the open air, and if possible some congenial regular mental occupation is important.

Without careful and persistent attention to these hygienic regulations, the most appropriate administration of medicines will fail to produce any permanent effect in the cure of epilepsy. To aid in overcoming the morbidly excitable condition of the nervous centres, we probably have no remedies more reliable than the bromides of potassium and ammonia, aided in some cases by gelsemin and in others by belladonna.

For the immediate relief of a convulsive paroxysm the bromide of potassium should be given in doses of 20 grs., or 30 grs. combined with 10 or 12 gtts. of tinct. belladonna, and repeated every two hours until the paroxysms cease. But for more permanent effect it should be continued steadily for a long period of time in doses of ten grains morning and noon, and fifteen grains at bedtime. The sedative influence in cerebral excitability will be increased by adding to each dose eight drops of tincture of gelsmium; or if there appears to be much tendency to vascular fulness of the cerebro-spinal centres the same quantity of tincture of belladonna may be substituted for the gelsmium. The female patient to which we have just directed your attention, affords nothing in her history calculated to explain the cause of her disease, except the fact that for a little time before the occurrence of the first paroxysm she had been separated from some of her relations and yielded to an inordinate degree of grief and despondency. Since her admission into the hospital she has taken 10 grs. of bromide of potassium before each meal and at bedtime, and has had no recurrence of the paroxysms, since the first twelve hours after her admission. We shall continue the same treatment, and as she is somewhat anemic we shall give her in addition one teaspoonful of the syrup of pyrophosphate of iron after breakfast and dinner each day. Before leaving this case, I cannot impress upon you too strongly the necessity of long continued perseverance in the treatment of epileptic cases. The patient and friends should be informed at once that no treatment will have any prospect of permanent success that is not continued faithfully, in all its details, for from six to eighteen months.

